# Evidence for the effects of prehabilitation before ACL-reconstruction on return to sport-related and self-reported knee function: A systematic review

**DOI:** 10.1371/journal.pone.0240192

**Published:** 2020-10-28

**Authors:** Florian Giesche, Daniel Niederer, Winfried Banzer, Lutz Vogt

**Affiliations:** 1 Division of Preventive and Sports Medicine, Institute of Occupational, Social and Environmental Medicine, Goethe University Frankfurt, Frankfurt/Main, Germany; 2 Department of Sports Medicine & Exercise Physiology, Institute of Sports Sciences, Goethe University Frankfurt, Frankfurt/Main, Germany; University of Belgrade, SERBIA

## Abstract

**Study design:**

Systematic review.

**Background and objectives:**

Preoperative neuromuscular function is predictive for knee function and return to sports (RTS) after reconstruction of the anterior cruciate ligament (ACL). The aim of this review was to examine the potential benefits of prehabilitation on pre-/postoperative objective, self-reported and RTS-specific outcomes.

**Methods:**

A systematic search was conducted within three databases. From the 1.071 studies screened, two randomized control trials (RCTs), two control trials (CTs) and two cohort studies (CS) met the inclusion criteria. Methodological quality rating adopted the PEDro- (RCT, CT) or Newcastle-Ottawa-Scale (CS).

**Results and conclusions:**

Methodological quality of the included studies was moderate (PEDro score: 6.5 ± 1.7; range 4 to 9). Two studies reported higher increases of the maximal quadriceps torque from baseline to pre-reconstruction: one study in the limb symmetry index (LSI), and one in both legs of the prehabilitation group compared to the controls. At 12-weeks post-reconstruction, one study (from two) indicated that the prehabilitation group had a lesser post-operative decline in the single-leg-hop for distance LSI (clinically meaningful). Similar findings were found in terms of quadriceps strength LSI (one study). At both pre-reconstruction (three studies) and two-year post-surgery (two studies), the prehabilitation groups reached significantly higher self-reported knee function (clinically meaningful) than the controls. RTS tended to be faster (one study). At two years post-surgery, RTS rates (one study) were higher in the prehabilitation groups. The results provide evidence for the relevance of prehabilitation prior to ACL-reconstruction to improve neuromuscular and self-reported knee function as well as RTS. More high quality confirmatory RCTs are warranted.

**Registration number:**

PROSPERO 2017: CRD42017065491.

## Introduction

Anterior cruciate ligament (ACL) reconstruction is the usual treatment for athletes after ACL tears, followed by evidence-based physical rehabilitation therapy to restore function [1, 2]. The final goal of the rehabilitation process after ACL reconstruction (ACLR), is to return to sport (RTS) to pre-injury level as quickly as possible without exposing the athlete at undue risk for re-injury [3, 4].

The RTS-decision should be based on the systematic and stepwise assessment of potential risk factors [5, 6]. Particularly, the use of clinical tests to assess an athlete’s neuromuscular function of the affected limb compared to the non-affected limb expressed by limb symmetry indices (LSI) appear to be a crucial criterion for RTS decision [7–9]. More symmetrical limb LSI are demonstrated to reduce the risk of reinjury [3, 10, 11]. Compared to healthy controls, individuals and athletes who returned to sport after primary ACLR show a up to six times higher incidence rate for re-injury within two years after surgery [12]. Furthermore, athletes who successfully returned to sports nevertheless often display a shorter career duration and an impaired game performance compared to controls after ACL reconstruction and RTS [13]. These issues highlight the importance of strategies for the improvement of the RTS-process.

One of these strategies is pre-operative rehabilitation (prehabilitation). Numerous studies indicate the relevance of preoperative neuromuscular performance factors, such as knee extension and flexion strength as well as single-leg-hop performance in ACL-injured individuals for postoperative knee function [14–19]. Accordingly, evidence-based guidelines for rehabilitation after ACLR also recommend pre-operative rehabilitation (prehabilitation) programmes with the aim to increase pre- and postoperative function [1].

Low-level evidence supports the relevance of prehabilitation to improve return to sports (RTS)-rates and two years self-reported knee function [20]. Prehabilitation appears to be effective for improving postoperative LSI of neuromuscular performance [16, 21]. Recently, a systematic Review conducted by Alshewaier et al. [22] indicates the positive value of preoperative training and post injury rehabilitation particularly in terms of increased knee-related function and improved muscle strength. As the authors of the review mainly included studies using a non-operative approach (no ACLR) and/or non-controlled trials (no usual care), the systematic assessment of potential benefits of prehabilitation on objective and self-reported outcomes before and after ACLR compared to usual care and its effects on RTS, is still lacking. Therefore, the aim of this systematic review was to examine the evidence for the effects of prehabilitation prior to ACLR and postoperative rehabilitation on pre- and postoperative RTS-specific neuromuscular outcomes, long-term self-reported knee functions and RTS-rates compared to ACLR and postoperative rehabilitation without prehabilitation.

## Materials and methods

The protocol for this systematic review was registered in the PROSPERO international prospective register of systematic reviews https://www.crd.york.ac.uk/PROSPERO/ (registration number: blinded for review). As studies on prehabilitation before ACLR often include participants below 18 years of age, the inclusion criteria on age was changed accordingly after the PROSPERO registration. This review is reported in accordance to the PRISMA statement.

### Study inclusion and exclusion criteria

We included randomized controlled trials, controlled trials (i.e. no randomized allocation into control and intervention groups) and prospective cohort studies published in English and German. We considered only studies including participants with primarily unilateral ACL rupture scheduled for reconstruction regardless of the surgical technique (i.e. single or double bundle technique), graft type (i.e. patellar or semitendinosus tendon) and concomitant injuries. Included studies had to assess, at least, one objective or subjective functional outcome measure and, at least, two of the following measuring points: Baseline (pre-prehabilitation), post-intervention (post-prehabilitation/ pre-surgery), post-surgery/after rehabilitation or follow-up.

Non-controlled studies (i.e. no usual care group) case reports, protocols, oral presentations and studies not based on original data were not included. Furthermore, trials including patients who were not scheduled for reconstructive surgery (non-operative rehabilitation), undergoing other orthopaedic operations than the reconstruction of the isolated, unilateral injured ACL, and scheduled for secondary ACLR were not included.

Intervention groups consisted of patients who received a preoperative exercise programme prior to ACLR and standard care following surgery (i.e. rehabilitation). Patients scheduled for ACLR who received standard treatment (usual care, i.e. no prehabilitation) before and standard care following reconstruction (i.e. rehabilitation) were considered controls.

### Database research

The databases PubMed/MEDLINE, Web of Knowledge and the Cochrane Library were searched using the following search terms:

*PubMed/Medline*: *(("acl"[All Fields]) OR "anterior cruciate ligament"[All Fields]) AND (Prehabilitation [All Fields] OR prehab [All Fields] OR pre-rehabilitation [All Fields] OR preoperative [All Fields] OR pre-operative [All Fields]) AND (exercise OR physiotherapy OR training OR intervention OR rehabilitation)*
*Web of Knowledge and the Cochrane Library*: *TOPIC*: *((("acl" OR "anterior cruciate ligament") AND (Prehabilitation OR prehab OR pre-rehabilitation OR preoperative OR pre-operative)) AND (exercise OR physiotherapy OR training OR intervention OR rehabilitation))*

The initial search was performed for studies published until October 31th, 2017; an update search was performed on June 12th, 2019.

Reference lists of the studies of interest were screened to identify extra articles. Two authors (FG, DN) independently selected trials for inclusion based on titles, keywords, and abstracts to determine eligibility. Any disagreements in terms of the study selection were discussed. If a conclusion could not be reached after discussion, a third reviewer (LV) was asked to resolve any conflicts. Full-texts of all trials considered eligible were retrieved. Then, one author (FG) performed first data extraction. Another author (DN) reviewed all data blindly. Again, any disagreements were discussed bilaterally. If a conclusion could not be reached, the third reviewer delivered the decisive vote. The following data from included papers were extracted: sample size, participant characteristics (diagnosis, number, age, sex and time since injury, if indicated), study methods (statistics), study specifics (setting, intervention, time points of assessment, and length of follow-up). Outcomes of interest were quadriceps strength and single-leg hop test (limb symmetry indices) as well as self-reported knee function. If not retrievable from the original publications, the authors were asked to send us the required data by e-mail.

### Study quality and risk of bias assessment

The methodological quality of (randomized) controlled trials was assessed using the PEDro scale (11 criteria). The PEDro scale is a valid and reliable tool to assess the methodological quality of clinical studies [23, 24]. The methodological quality of cohort studies was assessed by using the Newcastle Ottawa scale (NOS, 9 criteria) [25]. Two authors (FG, DN) independently assessed the risk of bias of the included (randomized) controlled trials using the Risk of bias tool described in the Cochrane Handbook version 5.1.0. We rated the risk of bias of the individual studies on an outcome-based level (self-reported and objective outcomes separately) according to the handbook. Outcomes were graded for risk of bias in each of the following domains: sequence generation, allocation concealment, blinding (participants, personnel, and outcome assessment), incomplete outcome data, selective outcome reporting, and other sources of bias. Risk of bias of the included cohort studies was rated using an assessment tool contributed by the CLARITY Group at McMaster University. Any disagreements were discussed. If a conclusion could not be reached after discussion, a third reviewer (LV) made the final decision.

## Results

### Search and synthesis of included studies

**Fig 1:**
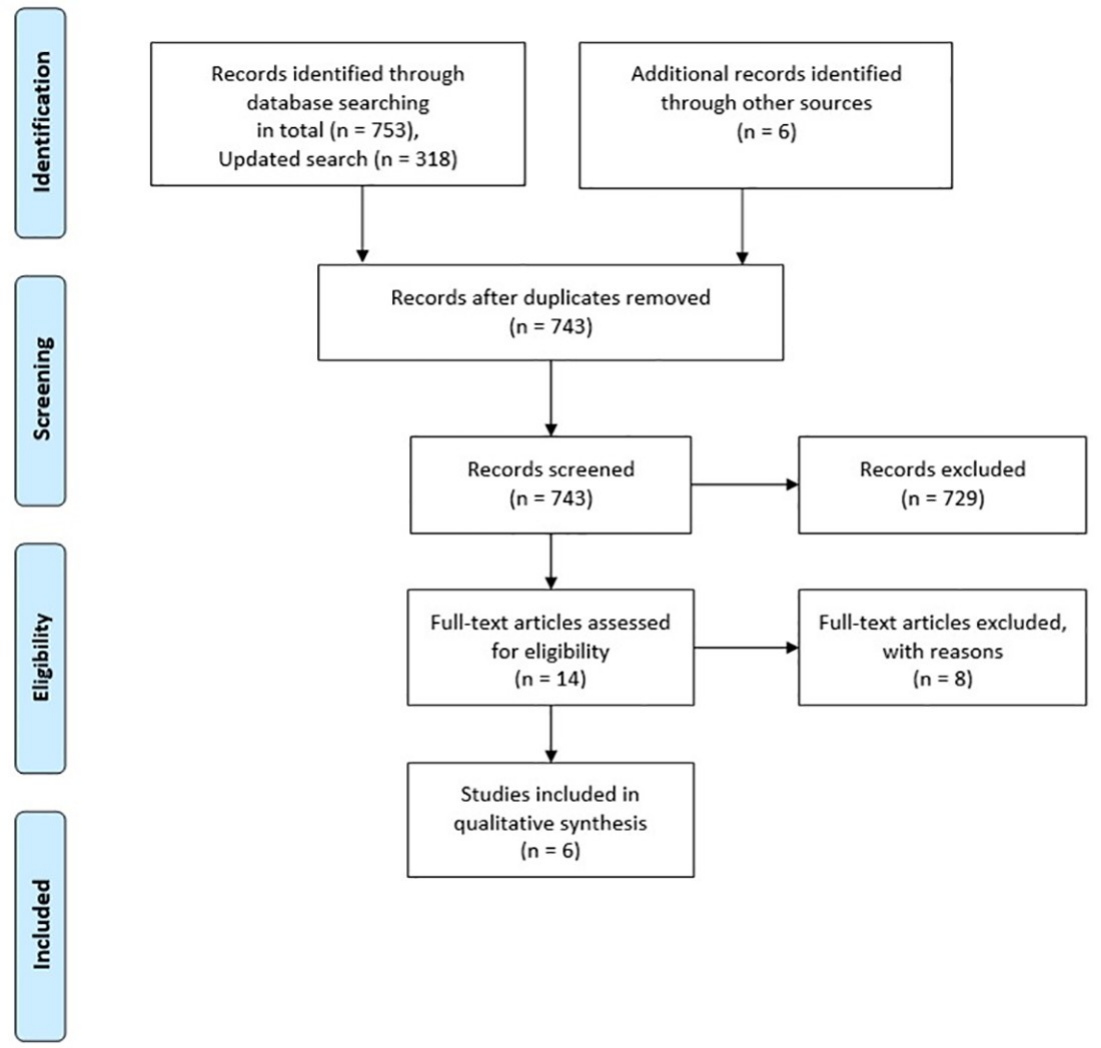
PRISMA flow diagram.

Fig 1 displays the research history and flow of the studies. From PubMed/ MEDLINE, we retrieved n = 674, from Web of Knowledge n = 305 and from The Cochrane Library n = 92 potentially eligible studies. Seven studies were excluded because they were 1) no controlled trials as both groups participated in a prehabilitation programme, or 2) used a non-operative rehabilitation approach (no ACLR) [16, 26–31]. One study was excluded, because it was a systematic review [22]. From the six trials included in qualitative analysis are two cohort studies [20, 32]; two controlled trials [33, 34] and two randomized controlled trials [35, 36].

### Methodological quality

According to the PEDro-Rating (11 points maximum), the controlled and randomized controlled trials were rated from four (one study [34]) over seven (two studies [33, 36]) to eight (one study [35]). The mean score of the studies was 6.5, which indicates an overall moderate methodological quality. The two cohort studies [20, 32] assessed with the NOS-scale displayed a fair to good quality (five [20] to seven [32] out of nine points).

#### Risk of bias

Risk of bias assessed for the controlled and randomized controlled trials was rated low for one study’s [35] (five out of seven), moderate for two studies’ [33, 36] (four out of seven points) and high for one studies’ outcomes [34] (two out of seven points) (Table 1). Risk of bias were mainly not rated different between the self-reported and objective studies’ outcomes (Table 1). In terms of the included cohort studies [20, 32], risk of bias was rated rather low for most items (Table 2).

**Table 1:** Risk of bias assessment of CT and RCT.

	Shaarani et al. [35]		Keays et al. [33]		Kim et al. [36]		Zdunski et al. [34]	
	self-reported	objective	self-reported	objective	self-reported	objective	self-reported	objective
Sequence generation	Low risk	Low risk	High risk	High risk	Low risk	Low risk	High risk	-
Allocation sequence concealment	Low risk	Low risk	High risk	High risk	unclear	unclear	High risk	-
Blinding of participants and personnel	unclear	unclear	unclear	unclear	unclear	unclear	unclear	-
Blinding of outcome assessment	Low risk	High risk	Low risk	Low risk	unclear	unclear	High risk	-
Incomplete outcome data	High risk	High risk	Low risk	Low risk	Low risk	Low risk	unclear	-
Selective outcome reporting	Low risk	Low risk	Low risk	Low risk	Low risk	Low risk	Low risk	-
Other potential threats to validity	Low risk	Low risk	Low risk	Low risk	Low risk	Low risk	Low risk	-
**Score**	**5**	**4**	**4**	**4**	**4**	**4**	**2**	**-**

**Table 2:** Risk of bias assessment of cohort studies.

	Grindem et al. [32]		Failla et al. [20]	
	self-reported	objective	self-reported	objective
Was selection of exposed and non-exposed cohorts drawn from the same population?	**Definitely yes (low risk of bias)**	-	**Definitely yes (low risk of bias)**	-
	Probably yes		Probably yes	
	Probably no		Probably no	
	Definitely no (high risk of bias)		Definitely no (high risk of bias)	
Can we be confident in the assessment of exposure?	**Definitely yes (low risk of bias)**	-	**Definitely yes (low risk of bias)**	-
	Probably yes		Probably yes	
	Probably no		Probably no	
	Definitely no (high risk of bias)		Definitely no (high risk of bias)	
Can we be confident that the outcome of interest was not present at start of study?	Definitely yes (low risk of bias)	-	Definitely yes (low risk of bias)	-
	Probably yes		Probably yes	
	**Probably no**		**Probably no**	
	Definitely no (high risk of bias)		Definitely no (high risk of bias)	
Did the study match exposed and unexposed for all variables that are associated with the outcome of interest or did the statistical analysis adjust for these prognostic variables?	Definitely yes (low risk of bias)	-	Definitely yes (low risk of bias)	-
	**Probably yes**		Probably yes	
	Probably no		**Probably no**	
	Definitely no (high risk of bias)		Definitely no (high risk of bias)	
Can we be confident in the assessment of the presence or absence of prognostic factors?	Definitely yes (low risk of bias)	-	Definitely yes (low risk of bias)	-
	**Probably yes**		**Probably yes**	
	Probably no		Probably no	
	Definitely no (high risk of bias)		Definitely no (high risk of bias)	
Can we be confident in the assessment of outcome?	Definitely yes (low risk of bias)	-	Definitely yes (low risk of bias)	-
	Probably yes		Probably yes	
	**Probably no**		**Probably no**	
	Definitely no (high risk of bias)		Definitely no (high risk of bias)	
Was the follow up of cohorts adequate?	**Definitely yes (low risk of bias)**	-	Definitely yes (low risk of bias)	-
	Probably yes		Probably yes	
	Probably no		**Probably no**	
	Definitely no (high risk of bias)		Definitely no (high risk of bias)	
Were co-interventions similar between groups?	Definitely yes (low risk of bias)		Definitely yes (low risk of bias)	
	**Probably yes**		**Probably yes**	
	Probably no		Probably no	
	Definitely no (high risk of bias)		Definitely no (high risk of bias)	

#### Participants characteristics

A total of 5.131 participants (thereof n = 4.961 from cohort studies) were included. Two studies recruited men only [35, 36]. The remaining four studies recruited both men and women. The mean age of the participants in the included trials ranged from 24.5 to 41 years (Table 1).

#### Participants`inclusion and exclusion criteria

Five studies [20, 26, 32, 42, 51] reported the inclusion of participants with primarily unilateral ACL rupture and one study included only individuals with chronic unilateral ACL ruptures [33] awaiting reconstruction. The clinical assessment of knee instability (i.e. pivot shift test, anterior drawer or Lachman test) was performed in two studies [33, 35], both with positive results. The remaining studies have not assessed knee stability. In one study recreational sports persons were included [35]. Two studies included only individuals who participated, at minimum, twice a week in jumping, cutting and pivoting sports such as football, basketball, American football, skiing or tennis, respectively performed these activities more than 50 hours per year before injury [20].

#### Exclusion criteria

Participants with any injury induced associated fractures, injuries to other ligaments in the same knee, collateral ligament injuries requiring repair/reconstruction and contralateral injuries [20, 32, 35, 36], full-thickness articular cartilage lesions [20, 32, 34], symptomatic meniscal injuries [20, 32], or previous injury or surgery of the involved or uninvolved knee [20] were excluded. Two studies specifically reported that only participants with isolated ACL rupture were included [35, 36]. One study has not reported any exclusion criterion [33].

#### ACL grafts

Bone-patellar tendon-bone (BPTB) grafts, Hamstring autografts and soft tissue allografts [20, 32] or BPTB grafts only [35] were used for reconstruction. The remaining three studies did not report the graft types used for reconstruction surgery [33, 34, 36].

#### Time before surgery/enrollment

In one study, the time from injury to surgery was 6.3 ± 4.1 months in the prehabilitation, and 6.8 ± 4.2 months in the rehabilitation cohort (control group) [32]. In another study, the mean time from ACL injury to reconstruction was approximately 5 months in the prehabilitation and 9 months in the control group [34]. One study stated that the time from injury to enrollment, which was 1.9 ± 1 months in the prehabilitation, and lower than 6 months in the rehabilitation cohort (control group) [20]. In another study, the average time from injury to baseline assessment was 6.7 months, ranging from 5 to 15 months [35], followed by a waiting time for surgery after baseline assessment of approximately 6 weeks. The remaining two studies have not reported any timeframes between injury and baseline assessment before reconstructive surgery [33, 36]. Further details on the study characteristics are displayed in Table 3.

**Table 3:** Characteristics for which data were extracted for each study included into qualitative and quantitative synthesis.

Study (year)	Participants (diagnosis, N analyzed, age (mean, SD), gender)	Measuring points /follow-up period	Treatment (n)	Control (n)	Outcomes	Statistics
Failla et al. (2016) [20]	Primarily unilateral ACL-R awaiting reconstruction; n = 2.187; 24,5 ± 9,5 years; 54,5% male	Baseline before (MOON) and after impairment resolution (DOC; before training) 2 years post reconstruction	*DOC-patients*: Prehabilitation, Rehabilitation (n = 192)	*MOON-Cohort*: Usual care, Rehabilitation (n = 1.995)	*Self-reported knee function* (IKDC, KOOS–subscales: Pain, symptoms, ADLs, sports/ recreation, QoL), RTS rates	*ANCOVA*: 2-year IKDC/ KOOS scores between groups (covariate: baseline IKDC/ KOOS scores)
						*ANOVA*: interaction of group and meniscal surgery/ graft types on 2-year IKDC scores
Grindem et al. (2015) [32]	Primarily unilateral ACL-R awaiting reconstruction; n = 2.774; 25,1 ± 7,5 years; 48,5% male	no baseline pre reconstruction 2 years post reconstruction	*NAR-patients*: Prehabilitation, Rehabilitation (n = 84)	*NKLR-Cohort*: Usual care, Rehabilitation (n = 2.690)	*Self-reported knee function* (KOOS–subscales: Pain, symptoms, ADLs, sports/ recreation, QoL)	ANCOVA:
						Comparison of KOOS in the two cohorts preoperatively and 2 years postoperatively (covariates: sex, age, time from injury to surgery, presence of cartilage and meniscus injury, Baseline KOOS) Stratification of preoperative KOOS subscale scores (Low/high scores were defined as scores below/above the median preoperative scores) Calculation of sex-specific KOOS cutoff points for each subscale to quantify the percentage of patients with KOOS within the normative range (18–34 yrs. age group)
Do Kyung Kim et al. (2015) [36]	Isolated ACL Rupture awaiting reconstruction; n = 80; 27.8 ± 5.7 years, 100% male	Baseline 3 month post reconstruction	Prehabilitation, Rehabilitation (n = 40)	Usual care, Rehabilitation (n = 40)	*Isokinetic knee strength (*range of 0 to 90° at an angular speed of 60°/ sec, with four repetitions) *sokinetic knee strength endurance* (angular speed of 180°/sec, with 20 repetitions)	Independent sample T-Tests:
						The highest peak torque value for each velocity was compared with the uninjured side (percent of strength deficit). For the single-leg hop test, the mean average distance was quantified by limb symmetry index (LSI) Repeated measures analysis to investigate the change in knee extensor strength and single-leg hop distance between groups
					→ therefrom derived *strength deficit* for each angular speed	
					SI of single-leg hop distance	
Keays et al. (2006) [33]	Chronic, unilateral ACL rupture awaiting reconstruction; n = 36; 29 ± 8 years; 69,4% male	Baseline Post training (pre reconstruction)	*ACLD-group 1*: Prehabilitation (n = 12)	*ACLD-group 2*: Usual care (n = 12)	*Knee joint stability* (KT1000 arthrometer which measured anterior translation on application of 67 Newton (N) and 89 N forces, and a manual maximum displacement test (MMDT); clinical stability assessed via anterior drawer, Lachman and pivot shift tests) *Quadriceps/ hamstring strength* at 60° and 120°/s via dynamometer single leg standing balance *Objective Functional performance* (agility: shuttle run, side-step and carioca tests) *Self-reported function* (via modified Noyes and Trust questionnaire)	ANOVA:
						2 x 3, side-to-side differences for strength, knee stability and balance were calculated (injured vs. uninjured side) Raw values were compared for the subjective questionnaires and the three agility tests. (Group matching for age, gender and activity level)
				*Healthy controls*: nothing (n = 12)		
Shaarani et al. (2013) [35]	Isolated ACL tear awaiting reconstruction; n = 23; 18–45 years (inclusion criteria); 100% male	Baseline Post-training (pre reconstruction) 3 month post reconstruction	Prehabilitation, Rehabilitation (n = 14)	Usual care, Rehabilitation (n = 9)	*Quadriceps and hamstrings strength* (average peak torque, average work per repetition, and deficits) assessed by use of isokinetic dynamometry (90°/s.) *Jump performance* (single legged hop test) *Pain and function* assessed via the Modified Cincinnati Knee Rating System score and Tegner activity level *RTS duration* (defined as return to preinjury levels of sport activities) *Quadriceps CSA* (via fMRI) CSA, MHC, mRNA, IGF-1, MuRF-1, MAFbx	ANOVA:
						One-way ANOVA was used to evaluate potential group differences in the baseline characteristics Mixed-design for repeated measures was used to analyze potential differences between groups over time
Zduński et al. 2015 [34]	Isolated ACL Rupture awaiting reconstruction; n = 30; 40 ± 8 years, 56.7% male	Baseline Post training (pre reconstruction)	Prehabilitation, Rehabilitation (n = 15)	Usual care, Rehabilitation (n = 15)	Self-reported knee function assessed by the Lysholm-Gillquist scale	Students t-Test and Mann-Whitney U tests were applied to detect differences from baseline to pre-surgery assessment within and between groups

Moon = Multicentre Orthopaedic Outcomes Network; DOC = Delaware-Oslo ACL Cohort; NAR = Norwegian Research Centre for Active Rehabilitation; NKLR = Norwegian Knee Ligament Registry; IKDC = International Knee Documentation Committee; KOOS = Knee injury and Osteoarthritis Outcome Score; ADLs = Activities of daily living; ACL = Anterior cruciate ligament; ACLD = anterior cruciate ligament-deficient; QoL = Quality of life; LSI = Limb symmetry index; CSA = Cross-sectional area; MHC = Myosin heavy chain; mRNA = messenger RNA; IFG-1 = Insulin-like growth factor 1; MuRF-1 = Muscle RING-finger protein-1; MAFbx = Muscle atrophy f-box.

### Intervention

#### Intervention vs. usual care (no prehabilitation)

All six studies compared preoperative exercise interventions with no treatment or usual care in participants scheduled for unilateral ACLR [20, 32, 33, 35, 36, 51]. In one study, usual care consisted of the maintenance of a normal daily physical activity level without any specific preoperative physical interventions [35]. In another trial [34], the participants of the control group received general recommendations and instructions on particular exercises for people with ACL tears. The remaining studies did not specifically define usual care [20, 32, 33, 36].

Postoperatively, all participants received a standardised, criterion-based rehabilitation programme aiming to increase knee RoM and weight bearing to restore gait pattern and limb symmetry of neuromuscular performance factors. In two studies, only preoperative measuring points were considered [33, 34].

#### Preoperative training interventions

Both prospective cohort studies [20, 32] did not describe any details about the training protocols used. Therefore, the authors referred to a study of Eitzen et al. [31].

*Type/content*. The preoperative interventions included strengthening exercises mainly of the lower limb in open and closed chain, [12, 16, 20, 23, 24, 32, 41, 50] neuromuscular (perturbation, balance, stability, proprioceptive exercises) training, [20, 32–36] muscle control and co-contraction exercises of the knee muscles with particular attention of the quadriceps [33, 34, 36], as well as stretching [33, 34] and RoM exercises [36] of the lower limb. One study included mobilization of the patella and used kinesiology taping of the patellofemoral joint [34]. Two studies included plyometric exercises, such as single leg hops with soft landing [20, 32].

*Intensity*. The same two studies used progressively increased strength training, with maximal effort for 3 or 4 sets of 6 to 8 repetitions. Progress was made, as the patients were told to perform as many repetitions as they could manage in the last three or four sets. If they were able to add two additional repetitions, weight was increased in the next session. Shaarani et al. [35] also used a progressive strengthening training approach including three sets of 12 repetitions with 10–15% weekly increase in the load. In the home-based exercise program of an ongoing study, strengthening and activation exercises of the lower limb muscles were conducted with 10 to 30 repetitions up to 30 to 50 repetitions, using materials such as elastic bands. Progression was made by increasing the number of sets. Stretching exercises of the lower limbs were performed about three sets with 30 seconds each [33], respectively RoM exercises such as knee extensions and flexions in sitting position for 10 minutes [36]. In one study, balance training, such as single leg standing on a balance board was performed about three sets with 30 secs each [36], 30 secs to 3 min with open eyes and 5–10 sec with closed eyes in another study. [33] Seperated in three consecutive phases, the 10 weeks perturbation protocol [31] performed by two studies [20, 32] contained a total of 10–12 sets of unilateral stance exercises on a rocker or roller board per session. To increase the level of difficulty, the participants executed additional movements of the arms or self-initiated perturbations [33].

*Duration/frequency*. The preoperative intervention lasted 4.8 weeks on average (range 4–6 weeks; [32, 34–36]. In one study, individuals participated in ten neuromuscular training sessions before ACL [20]. One study did not report information about the length of the intervention period [33]. The training frequency ranged from two to four times per week [20, 32, 34, 35] with a duration of a maximum of 75 to 120 minutes [20, 32, 34], including 10- to 20-minute warm-up exercises on a stationary ergometer cycle [20, 32, 36]. One study reported daily home-based training of 30 minutes’ duration [33].

*Supervision/setting*. In three studies, the prehabilitation group was supervised twice a week with two additional home-based training sessions a week [20, 32, 35]. The participants were supervised, at least, three times a week in one study [36], but the authors of this study did not provide information whether additional home-based training sessions were required. The supervised training took place in a hospital [36] or gym [35]. A completely home-based training approach was used in one study [33].

*Training compliance and dropouts*. According to the study of Shaarani et al. [35], three participants in the prehabilitation group did not complete the program because of time constraints. All patients in the exercise group completed more than 90% of the exercise protocol. In another study, participants who participated in a home-based training programme, reported high levels of compliance with a minimal self-score rating of 8 out of 10 [33]. None of the remaining four studies have reported dropout rates, nor reported any information about training compliance.

### Pre-prehabilitation / baseline to pre-surgery effects

#### Objective outcomes

Two studies investigated the effects of prehabilitation on the limb symmetry index of quadriceps strength from baseline to pre-reconstruction. Keays et al. [33] observed a significant increase of the limb symmetry index from 85% to 102% (60°/s, p < .05, d = 1.7) and from 86% to 103% (120°/s, p < .05, d = 1.6) in the prehabilitation group. No significant changes occurred in the usual care and the healthy control group (p > .05). Shaarani et al. [35] observed significant increases of the quadriceps peak torque (90°/s) in both the injured and uninjured limbs compared to baseline in the prehabilitation group only (p < 0.05). Nevertheless, the peak torques did not differ systematically between groups neither at baseline nor at pre-surgery (p > .05) (Table 4). Contrary to Keays et al. [33], Shaarani et al. [35] found no significantly higher increases of the limb symmetry index in the prehabilitation (+3.2%) relative to the control group (+6.6%) (based on original data sent by the authors).

**Table 4:** Descriptive results of the individual studies included into qualitative and quantitative synthesis.

Study (year)	Results
Failla et al. (2016) [20]	*Baseline*:
	•No differences between groups in age, sex, or body mass index
	•Significantly higher proportion of concomitant meniscal surgery performed (p = .029) in the MOON cohort
	•DOC patients had significantly higher baseline IKDC compared to MOON cohort (70 ± 13 vs. 50 ± 17; p < .001), which exceeded the MCID
	•The preoperative training group had significantly higher baseline KOOS values across all subscales than MOON cohort patients (Pain: 84 vs. 73, Symptoms: 75 vs. 67, ADL: 93 vs. 82, Sports/Recreation: 66 vs. 48, Quality of Life: 51 vs. 37).
	*2-years IKDC scores*:
	•After controlling for baseline IKDC scores, DOC patients continued to have significantly higher IKDC scores than MOON cohort (84 ± 25 vs. 71 ± 32; p < .001)
	•Post hoc power analysis revealed the ability to detect a difference of 2 points on the IKDC between groups
	•No significant group x meniscal procedure (p = .345) or group x graft type (p = .073) interactions on 2-year IKDC scores
	*2-years KOOS-subscale scores*:
	•After controlling for baseline KOOS values, DOC patients continued to have higher and clinically meaningful differences across all KOOS subscale scores compared with MOON cohort (Pain: 94 vs. 78, Symptoms: 89 vs. 72, ADL: 98 vs. 82, Sports/Recreation: 85 vs. 70, Quality of Life: 76 vs. 64).
	*2-years RTS-rates*:
	RTS rates were significantly higher in the DOC compared with MOON cohort (p < .001)
Grindem et al. (2015) [32]	*Preoperative KOOS-subscale scores*:
	•No significant differences between the two cohorts in age, sex, time to surgery, presence or severity of cartilage or meniscus injuries
	•NAR-patients had significantly better preoperative KOOS in all subscales (differences in all subscales except Symptoms were clinically relevant): Pain: 87 vs. 75.9, Symptoms: 82.6 vs. 73.6, ADL: 94.7 vs. 85.1, Sports/Recreation: 69.1 vs. 45.2, Quality of Life: 49.6 vs. 36).
	*2 years KOOS-subscale scores*:
	•NAR cohort still showed significantly better KOOS in all subscales, and clinically relevant differences were found in KOOS Symptoms, Sports and QoL (largest group differences again for KOOS—Sports; 17.7 points)
	•After controlling for the preoperative KOOS, the NAR cohort had significantly better KOOS scores (Pain: 93.5 vs. 86, Symptoms: 89.2 vs. 77.4, ADL: 98 vs. 92.5, Sports/Recreation: 85.1 vs. 67.6, Quality of Life: 78.6 vs. 67.7).
	•In patients who had preoperative scores below the median score, the NAR cohort showed 20.6 higher KOOS—Sports scores (p = .003), and 12.3 points higher KOOS—QoL scores (p = .006)
	•A higher percentage rate of patients in the NAR cohort scored within the normative range in the different KOOS subscales compared to NKLR-cohort
Do Kyung Kim et al. (2015) [36]	•Patients of IG showed a significantly lower post-operative loss of knee extensor strength deficits both at an angular velocity of 60°/s (Prehab: 22.8±13.7 to 28.5±9.0, p = .018), and 180°/s (16.6±10.6 to 23.3±9.0, p = .033) compared to CG (60°/s: 23.5±15.8 to 36.5±10.7, p > .05; 180°/s: 17.5±11.9 to 27.9±12.6, p > .05).
	•The IG also showed significant improvements in the single leg hop for distance test (higher limb symmetries; p = .029) in comparison to CG.
Keays et al. (2006) [33]	*Comparison between groups (Baseline)*:
	•No significant differences in any measure existed between the two injured groups (Exception: hamstring strength measured at 60°/s).
	•Significant differences in all measures between each injured group and the control group (Exception: hamstring strength measured at 120°/s, eyes-open and foam balance tests).
	*Comparison between groups (post-training)*:
	•Significant differences existed between the treated (Group T) and untreated (Group NT) injured groups for quadriceps strength (p < .001), standing balance measure, for the three agility measures (p = .002; p = .003; p = .001) and for the Noyes and Trust questionnaires (p < .001 for both).
	•No differences existed between the treated and healthy, control group (Group C) for quadriceps and hamstring strength, balance measures or agility measures (p>.05). However, differences still existed for objective knee joint stability testing and for subjective testing.
	•Differences between the untreated group (Group NT) and control group (Group C) remained unchanged.
	*Time effects (Baseline to post-training)*:
	•Significant improvements in quadriceps strength for Group T (p < .01) from strength indices (60°/s: 0.85 to 1.02; 120°/s: 0.86 to 1.03) compared to NT (60°/s: 0.74 to 0.75; 120°/s: 0.85 to 0.81) and C (60°/s: 1.01 to 0.99; 120°/s: 1.04 to 1.05).
	•No significant improvements of Group T, NT and C in terms of hamstring strength.
	•Significant decrease in side-to-side translation measured at the 89 N testing force in Group T (p < .003)
	•Balance improved significantly in Group T for eyes-closed (p < .001) as well as for eyes-open (p = .036)
	•Group T improved significantly in all agility measures (p < .05)
	•Group T only demonstrated significant improvements in scores for both the Noyes (57±14 to 70±6, p < .05, d = 1.1) and Trust (4.7±3.1 to 8.3±2.9, p < .05, d = 1.2) assessments (p < .001)
	•No significant changes in the other both groups (exception Group NT balance had worsened; p = .002)
	*Group x Time interactions*:
	•Were found for quadriceps strength (improved limb symmetries) at 60°/s (p < .001) and 120°/s (p < .001), for knee joint stability (p = .041), for standing balance with eyes-open (p = .002) and eyes closed (p = .006; F = 6.13) and foam balance (p = .042), for functional performance such as the shuttle-run (p = .001), the side-step (p = .021), the carioca test (p = .004) and subjective function such as the Noyes score (p < .001) and the Trust score (p < .001)
Shaarani et al. (2013) [35]	*Single leg hop performance (before surgery and at 12 weeks’ post-reconstruction)*:
	•The single-legged hop test results improved significantly in the injured limb compared with baseline (p = .001). Mean single leg-hop test scores were higher preoperatively in the exercise group than the control group (p = .001).
	•At 12 weeks postoperatively, the rate of decline in the single-legged hop test was reduced in the exercise group compared with control (p = .001).
	*Quadriceps peak torque* (*before surgery and at 12 weeks’ post-reconstruction*):
	•Quadriceps peak torque increased significantly with similar gains in CSA in both the injured (p = .001) and uninjured limbs (p = .009) after prehabilitation compared with baseline.
	•However, there was a significant decrease in quadriceps peak torque of the injured limb in the exercise group at 12 weeks postoperatively compared with baseline (p = .042) and preoperative time points (p < .001). No statistically significant differences between both groups for the injured limbs at any time point.
	*Hamstrings peak torque* (*before surgery and at 12 weeks’ post-reconstruction)*:
	•Compared with baseline, preoperative hamstring peak torque increased significantly in the injured limb in both the exercise (p = .034) and control group (p < .001). No significant differences were seen between the exercise and control groups at both pre- and postoperative time points.
	*Cincinnati scores (before surgery and at 12 weeks’ post-reconstruction)*:
	•The mean modified Cincinnati scores were increased significantly from baseline to pre-operative and to 12 weeks postoperative time points in the exercise group only (p = .004; p = .001). There was a significantly higher mean score (p = .004) in the exercise group compared with the control group only at 12 weeks postoperatively.
	*RTS-duration*:
	•The mean time to return to sport was shorter for the control and exercise group. The difference almost reached statistical significance (p = .055).
Zduński et al. 2015 [34]	The self-reported knee function (Lysholm score) improved in both groups. At pre-prehabilitation, patients from the prehabilitation group reported poor knee function. At the pre-surgery measurement time point, the mean score had increased significantly. The difference was statistically significant (p < .001). At pre-prehabilitation, the control group reported significant higher self-reported knee function than the prehabilitation group. At the second measurement, directly before the ACL-reconstruction, the mean score improved. However, a greater pre-post improvement of the injured knee joint was found in patients from the prehabilitation group.

Moon = Multicentre Orthopaedic Outcomes Network; DOC = Delaware-Oslo ACL Cohort; NAR = Norwegian Research Centre for Active Rehabilitation; NKLR = Norwegian Knee Ligament Registry; IKDC = International Knee Documentation Committee; KOOS = Knee injury and Osteoarthritis Outcome Score; Group T = Injured group receiving preoperative physiotherapy treatment; Group NT = Injured group receiving no preoperative physiotherapy treatment; Group C = Uninjured control group; IG = Intervention group; CG = Control group; MCID = Minimal clinically important differences; ADL = Activities of daily living; QoL = Quality of life; CSA = Cross-sectional area.

One study [35] measured the single-leg hop for distance performance: Compared to the control group, the authors found higher increases of the single-leg hop scores of the injured limb in the prehabilitation (13.5%) compared to the control group (9%), resulting in an overall preoperative score of 183.6 ± 16 in the prehabilitation and 156 ± 43 in the control group. These improvements were significant (p < .05) in the prehabilitation group only. However, both groups did not differ significantly neither at baseline nor at pre-surgery (p > .05).

One study [33] assessed knee joint stability, balance and agility. The authors found significantly higher improvements in each of these outcomes for the prehabilitation compared to the control groups (Table 4). In terms of hamstring peak torque assessed by two studies [33, 35], no significant advantage was found for the prehabilitation group at pre-surgery (Table 4).

#### Self-reported outcomes

Two studies [33, 34] examined the effects of prehabilitation on pre-operative self-reported knee function (Noyes, Trust [33] and Lysholm score [34]): Significant higher improvements were found for the prehabilitation compared to the control groups for both the Noyes (d = 1.1) and Trust (d = 1.2) scores (Table 4). In terms of the Lysholm score, Zdunski et al. [34] reported a mean pre- to post change from 46 to 66 points in the prehabilitation and from 59 to 64 points in the control group. Similar findings were gained by another study [35]: The authors observed a significant increase of the mean Cincinnati score (62.6 to 76.5 vs. 66 to 70 points) in the prehabilitation group only. At pre-surgery, both groups did not differ significantly.

### Pre-prehabilitation or baseline to post-rehabilitation (12-week after surgery)

#### Objective outcomes

Two studies [35, 36] examined the effects of prehabilitation on quadriceps strength at 12-weeks post-surgery. Kim et al. [36] observed a significant lower post-operative loss of the limb symmetry of the knee extensor strength relative to baseline in the prehabilitation compared to the control group at both an angular velocity of 60°/s (-5.7% vs. -13%) and 180°/s (-6.7% vs. -10.4%) (Table 4). This resulted in a more symmetric index at both 60°/s (prehab: 28.5 ± 9.0 vs. control: 36.5 ± 10.7, p < .05, d = 0.8) and 180°/s (23.3 ± 9.0 vs. 27.9±12.6, p < .05, d = 0.4) in the intervention group. Similarly, Shaarani et al. [35] found a trend for a lower reduction of the baseline limb symmetry index in the prehabilitation compared to the control group (-20.3% vs. -24.8%, p > .05; based on original data sent by the authors).

Two studies [35, 36] investigated the effects of prehabilitation on the single-leg jump performance from baseline to 12-week post-reconstruction. Kim et al. [36] reported a significant increase of the limb symmetry index for the prehabilitation (75.1 to 85.3%, p < .05, d = 1.1), but not for the control group (76.5 to 80.5%, p > .05, d = 0.4). Shaarani et al. [35], found a reduction of the single-leg jump test scores of the injured limb relative to pre-surgery in both groups (p < .05). Nevertheless, the prehabilitation group maintained a higher score compared to the control group (144.9 ± 15.5 vs. 113.3 ± 25.5, p < .05, d = 1.5). Relative to the controls, the prehabilitation group indicated a lower reduction of the baseline limb symmetry (-10.8% vs. -17.6%, p > .05; based on original data sent by the authors). However, these changes were not significant.

In terms of hamstring peak torque assessed by one study [35], no significant advantage was found for the prehabilitation group (Table 4).

#### Self-reported outcomes

One study [35] examined the effects of prehabilitation on 12-weeks postoperative self-reported knee function: the mean modified Cincinnati scores increased significantly from baseline to 12-weeks post-surgery (prehab: 62.6 to 85.3, p < .05; controls: 66 to 77.6, p > .05) resulting in a significant higher mean score for the prehabilitation compared to the control group (85.3 vs. 77.6, p < .05).

### Two-year self-reported knee function and return to sport

Two cohort studies [20, 32] compared the level of self-reported knee function ((International Knee Documentation Committee, Knee injury and Osteoarthritis Outcome Score)). Both studies indicated a superior effect of prehabilitation, when compared to usual care. In both studies, the prehabilitation cohort exhibited significantly higher baseline/preoperative [20, 32] scores than the controls. Controlled for this confounder, the prehabilitation cohort continued to have significantly higher Knee injury and Osteoarthritis Outcome Score values in all subscales [20, 32] and International Knee Documentation Committee [20] scores (84 vs. 71) at 2-years post-surgery (Table 4).

The RTS duration and rates were assessed by one study in each case [20, 35]. There was a trend for significant faster RTS of the prehabilitation compared to the control group 34.18 ± 4.14 vs. 42.5 ± 10.46 weeks, p = .055) [35]. Nevertheless, no re-injuries occurred in the prehabilitation group during a follow-up period of 15 month after reconstruction [35]. According to another study a significantly higher share of participants from the prehabilitation compared to the control group returned to their preinjury sport at the two years’ follow-up [20] (72% vs. 63%; p < .05).

## Discussion

Low to moderate quality evidence indicates that exercises have a positive impact on pre-operative and postoperative functional performance. Low-level quality evidence supports the superiority of prehabilitation in terms of self-reported knee function at both pre-reconstruction and three months as well as two years after ACLR. The results further provide indications for higher RTS rates and a trend for a shorter time until RTS through prehabilitation.

### Quadriceps strength limb symmetry index

Superior intervention effects on quadriceps peak torque LSI were found preoperatively by one study [33], and at three months’ follow-up by another study [36]. In contrast, Shaarani et al. [35] reported no significant effects.

The latter findings [35] may be attributed to the fact that the maximum quadriceps strength improved significantly for both the injured and uninjured limb after prehabilitation. These symmetric training-induced improvements may have led to the maintenance of the already existed baseline asymmetries between both limbs. Contrary, in the study of Keays et al. [33], prehabilitation may have resulted in a disproportionate higher preoperative improvement of the quadriceps strength of the injured relative to the unijured limb. Possibly, the prehabilitative intervention in the study of Keays et al. [33] was more specific to quadriceps strength of the injured limb (unilateral strength training) as the intervention by Shaarani et al. [35]. Furthermore, Keays et al. [33] encouraged their participants to perform daily home-based exercises while Shaarani et al. [35] used a gym- and home-based approach with four training sessions per week. However, the influences of the intervention period (6 weeks) and content (strength and balance training) on the treatment effects can be considered low, as both studies were comparable in this regard. An alternative explanation for the higher preoperative LSI observed by Keays et al. [33] may be that the quadriceps peak torque of uninjured limb decreased relative to the injured limb over the preoperative period as a potential result of a lower physical activity level after injury. This mutual approach of both limbs may have resulted in a higher LSI. According to Wellsandt et al. [37], this may consequently lead to an overestimation of the neuromuscular performance of the injured limb.

The conservation of the preoperative neuromuscular performance (lower loss) through prehabilitation beyond the postoperative rehabilitation period may explain both the continuously higher symmetry indices of the prehabilitation compared to the control group indicated by Kim et al. [36] and the still non-significant different limb symmetries between both groups indicated by Shaarani et al. [35] at 12-weeks postoperative.

### Single-leg hop for distance limb symmetry index

Kim et al. [36] found an increased single-leg hop for distance LSI in the prehabilitation compared to the control group at three months after ACLR relative to baseline. According to Reid et al. [38] the improvements of the prehabilitation group exceeded the level for a minimal detectable change (MDC: 8.1%). In Shaarani et al. [35], the LSI was reduced in both groups after postoperative rehabilitation compared to baseline. However, there was a trend for a lesser decline for the prehabilitation compared to the control group. The lower decline in the prehabilitation relative to the control group did not completely meet the cut-off for a minimal detectable change [38]. Nevertheless, the prehabilitation group maintained the higher pre-operative single-leg hop performance.

Single-legged hop tests are highly reliable in ACL-injured and -reconstructed participants [29, 38]. The single leg hop for distance is a valid and reliable performance-based outcome measure reflecting the combination of leg strength, neuromuscular control and self-confidence in the ACL reconstructed knee [38], as well as the ability to tolerate sports-specific loads [39]. Thus, it may be possible that prehabilitation fostered the restoration of mechanical stability, and patients’ confidence, in their knee stability. Consequently, the potentially reduced fear of re-injury may have had positive implications on the postoperative single-leg hop for distance LSI shown by both studies (high effect sizes). Nevertheless, these findings underline, particularly, the specificity of the used prehabilitation contents to jump ability and neuromuscular control. Together with the quadriceps strength, the single leg hop for distance was recently shown to be of prognostic value of an ACL re-injury [10].

### Self-reported knee function

Beneficial effects of prehabilitation on both preoperative [33, 34] and two year postoperative self-reported knee function [20, 32] were reported. In terms of the Lysholm score, the improvements in the prehabilitation group from baseline to pre-surgery exceeded the cut-off for the minimal clinical important difference (10 to 17 points [40]). Regarding the Cincinnati knee score, the increases of the prehabilitation group from baseline to pre-surgery and to three month after ACLR met or even exceeded the minimal clinical important difference of 14 points [41] in contrast to the control group. The higher International Knee Documentation Committee scores at both baseline (70 vs. 50 points) and two years post-surgery (84 vs. 71 points) of the prehabilitation compared to the control group exceeded the cut-off for minimal detectable change (8.8 to 15.6 [42]). Similar findings occurred for the Knee injury and Osteoarthritis Outcome Score: At two years post-surgery, the prehabilitation continued to exhibit higher values as the control group, which exceeded the minimal detectable change in almost all subscales (pain: 6–6.1, symptoms: 5–8.5, activities of daily living: 7–8, sports/recreation: 5.8–12, quality of life: 7–7.2% [42]).

These positive implications may be a consequence of the prehabilitation-related improved pre- and postoperative neuromuscular performance as indicated above. The assumed association between objective and self-reported outcomes are supported by Logerstedt et al. [39] providing evidence for the predictive value of the 6 months’ postoperative single-legged jump performance for the self-reported knee function one year after ACL surgery. Furthermore, evidence suggests the predictive value of preoperative neuromuscular performance for postoperative self-reported function and RTS [14–19, 43].

### Return to sport time points and rates

The higher RTS rates or shorter time until RTS success found by two studies [20, 35] may be a consequence of the improved postoperative objective and self-reported knee function of the prehabilitation groups as indicated above. The observed RTS rates were, in both the intervention and control groups (in particular in the latter one) slightly below but comparable to such reported in other studies. According to a systematic review of Ardern et al. [44], 81% of the ACL-reconstructed individuals returned to some kind of sports, 65% returned to their preinjury level and 55% returned to competitive sport.

### The prehabilitation programs and their practicability

The prehabilitation programmes of the included studies differed in terms of frequency, intensity, time, supervision and setting. Nevertheless, the preoperative training protocols varied less in terms of content: The studies included in the review primarily adopted stretching and balance exercises as well as strengthening and control and co-contraction training with particular focus on the quadriceps. The prehabilitation protocols also included hamstrings strengthening. The hamstring muscles represent a major synergist of the ACL as its contraction reduces anterior tibia translation [45]. In the included cohort studies [20, 32], preoperative treatment was subdivided into two phases. The goal of the initial phase (up to two months after injury) was to resolve inflammatory symptoms and to restore full knee ROM [31]. After impairment resolution (about two month after surgery in average) [16, 20], a 5-week progressive exercise program (second phase) was performed aiming to restore muscle strength and neuromuscular function using intensive muscle strength, plyometric, and advanced neuromuscular exercises. [31]

Based on two of the included studies training compliance appears to be high and dropout rates appear to be low [33, 35]. Therefore, prehabilitation seems to be safe and feasible in participants with ACL injury. This is supported by Eitzen et al. [31]. The authors found high compliance to and tolerance for early staged interventions after ACL injury.

### Time between rupture and reconstruction

The average time from injury to surgery 6.5 months [32], 8.2 months [35] and 5 to 9 months [34]. In the remaining two studies, the preoperative timeframes were not reported, but Keays et al. [33] described the inclusion of individuals with chronic ACL deficiencies, which also implies rather longer periods between injury and surgery.

Due to the relatively long preoperative period, some participants may have been engaged in physical training and exercise before or beyond prehabilitation. Except both of the cohort studies in which exercises were already performed early after injury to restore basic knee function, no of the other studies reported if and which exercises/treatment preceded prehabilitation. Therefore, we cannot estimate the effects of these preoperative actions on the results of this review.

### Methological quality and limitations of the included studies

A common limitation in exercise trials is the limited possibility to blind participants [46]. This limitation may particularly lead to a biased assessment of self-reported knee function. In order to reduce the risk of bias as much as possible, all investigators shall be blinded to intervention allocation. This was not reported in most of the included studies. The heterogeneity of the study population may further limit the possibility to derive specific practical relevance. Together, with the (partially) randomised study designs and the consideration of known and suggested confounders, the included studies may provide a sufficient statistical power.

However, the included studies have certain limitations:

- Time between diagnosis of ACL tear and surgical reconstruction was not consistently reported across the included studies.
- No consistent definition of standard care treatment (control condition) was provided. Physical activities and therapies potentially performed early after ACL injury have not been reported neither for interventions nor for control groups.
- The heterogeneity across studies in terms of the characteristics of participants was high. For instance, the age of the participants included in one study was quite high (about 40 years) relative to the age groups (18 to 25 years), which are mainly affected by ACL-injuries [47]. The kind of sport and physical activity levels/demands were not described in most studies and one study included only those with chronic ACL deficiency [33].
- Methodological quality of the included studies was moderate and risk of bias ranged from low to high.

### Limitations of this review

This systematic review has also certain limitations:

- We included only studies written in English and German. Therefore, relevant literature published in other languages may not have been included.
- The number of the studies included into this systematic review was quite low (n = 6).
- The evaluation of the effects of prehabilitation on long-term self-reported function is based on two prospective cohort studies implying low levels of evidence. Furthermore, both studies referred to the same prehabilitation cohort. The partial overlap of participants may have resulted in a substantial bias. Furthermore, the prehabilitation group exhibited higher self-reported knee function at baseline. Although considered as a covariate in statistical analyses, this may have limited the comparability between both groups.
- Due to the small number of studies available investigating the same outcome at comparable measuring points, the overlap in participants in two studies, the diverse and partly unknown content of the interventions, as well as the variations in study populations, we did not performed qualitative data syntheses (meta-analyses).
- We only screened the databases PubMed (Medline), Web of Knowledge and the Cochrane Library. Considering the topic of our review, almost all manuscripts of interest should be found therein. However, expanding the search to even more databases, like EMBASE, PEDro, CINAHL, AMED, SportDiscus and CENTRAL may would have led to slightly more hits.

## Clinical implications

Our findings apply to physically active adults with primary, unilateral ACL rupture without additional severe injuries to other intraarticular structures who had delayed ACLR. Besides improved preoperative functions, this review provides first evidence that prehabilitation may reduce the decline of postoperative neuromuscular performance of the lower limb and improve self-reported knee function and RTS success. Although more high quality confirmatory RCTs are needed to finally evaluate the clinical importance of prehabilitation, the outcomes found to be impacted by prehabilitation in this review are mostly the same as those found to be predictive for a second ACL injury [10]. Despite these positive implications, the majority of the orthopaedic surgeons seems to consider prehabilitation less important in the preoperative care of ACL-injured individuals [48].

Delayed compared to early ACLR is unlikely to result in postoperative differences in secondary knee pathologies (incidence of meniscal/ chondral lesions, postoperative infection, graft rupture) and functional outcomes [49] as well as two year self-reported knee function [27]. Against this background, it appears plausible that additional preoperative training prior to a delayed surgery may result in better postoperative function compared to early surgery.

Furthermore, the pre-ACLR contact time associated with a delayed surgery can help to identify ACL-deficient individuals without knee instability who may be able to RTS without surgery (copers [50]). This is in line with previous findings, which indicated that, more than half the ACLR can be avoided without aversively affecting outcomes using a delayed ACLR approach [27]. In line with current work applicable in non-professional or leisure time copers [49], delaying surgery for a minimum of 3 months after the ACL tear may be recommended [51].

Based on the studies included in this review, the preoperative or post-injury training protocols (4 to 6 weeks, 2 to 4 times per week) should contain muscle control and co-contraction exercises of the knee muscles with particular attention of the quadriceps as well as strengthening (open and closed chain) and stretching exercises of the lower limb. Moreover, advanced neuromuscular (perturbation, balance, stability, proprioceptive exercises) as well as plyometric exercises (e.g. single leg hops with soft landings) need to be considered. Such more intensive preoperative interventions should be not started before initial impairment resolution (about 2–3 months) in dependence on the functional and tissue repair status as well as concomitant injuries. Although, there are no specific evidence-based guidelines for strength and neuromuscular training in the early stage after ACL injury available yet, we recommend in exemplary the exercise protocols published by Eitzen et al. [31] and Wilk et al. [52] as a guidance.

## Conclusion

Low to moderate quality evidence indicates that exercises have a positive impact on pre-operative and postoperative functional performance. Low-level quality evidence supports the superiority of prehabilitation in terms of self-reported knee function at both pre-reconstruction and three months as well as two years after ACLR. Due to the low number of studies included into this systematic review, divergent methodologic quality and high heterogeneity across the studies, high quality RCTs are warranted to finally evaluate the clinical importance of prehabilitation. Specifically, future trials need to investigate, if prehabilitation before delayed compared to early ACLR lead to a faster or improved restoration of postoperative RTS-specific neuromuscular or self-reported function and sport participation. Furthermore, the potential effects of prehabilitation on re-injury incidences need to be assessed.

## Supporting information

S1 Checklist (DOC)
